# Editorial: Augmented-, virtual- and mixed reality in visceral surgery

**DOI:** 10.3389/fsurg.2023.1305757

**Published:** 2023-10-18

**Authors:** Dirk Weyhe, Verena Nicole Uslar, Tim Schneider

**Affiliations:** Department for Human Medicine, University Clinic für Visceral Surgery, Pius-Hospital Oldenburg, University Medicine Oldenburg, Oldenburg, Germany

**Keywords:** virtual environment, AR, VR, XR, surgical education, surgical planning and simulation

**Editorial on the Research Topic** Augmented-, virtual- and mixed reality in visceral surgery

## Introduction

Techniques such as Augmented Reality (AR), Virtual Reality (VR), and Mixed Reality (MR) are increasingly entering the medical workplace and, in conjunction with the field of visceral surgery, represent a potential paradigm shift that could reshape surgical strategies, training methods and patient education. For this reason, the present research topic serves to explore the interrelationship between these immersive technologies and the complex field of visceral surgery.

The development of patient-specific, three-dimensional organ models using advanced imaging modalities such as computed tomography (CT) and magnetic resonance imaging (MRI) had enabled groundbreaking progress in this field, offering immense potential of immersive technologies in the field of visceral surgery.

Enhanced by AR or embedded in a virtual environment by VR, these complex, patient-specific 3D models provide surgeons with unprecedented insight into the complexity of their patients’ condition, potentially improving not only the precision of surgical planning but also providing an excellent opportunity for patient education. Furthermore, it provides surgeons, both experienced and trainee, with the unique ability to immerse themselves in immersive training scenarios that mimic real-world situations. These capabilities will allow these technologies to serve as cornerstones for the next generation of surgeons to train their skills in a risk-free environment.

The collaborative nature of this research topic embodies the multi-faceted methodology essential for navigating the unexplored terrain of Extended Reality in the context of visceral surgery. This field brings together general surgeons, visceral surgeons, anatomists, biologists, and distinguished faculty members from renowned medical institutions. Their common effort is to advance the clinical, translational, and basic research that provides the foundation for these new technologies. Through the combined effect of our efforts, we aim to uncover the full spectrum of the potential of AR, VR, and MR, thereby finding solutions to both known and unforeseen challenges.

In this context, Cetin et al. particularly address the utilization of a VR headset as a tool to assess the feasibility of an innovative illumination concept for the operating room. Meanwhile, Reinschlüessel et al. and Staubli et al. focus on the 3D visualization of patient-specific anatomical structures and the utilization of the generated models for preoperative planning. In addition, Pu et al. showed the possibilities of using SpyGlass in the treatment of complex pancreatic stones of the pancreatic duct.

## Papers in the research topic

Cetin et al. describe a feasibility study regarding the utilization of automated lighting systems in the operating room. Both real-world and virtual tests were conducted to investigate the effectiveness of the automated lighting systems. The findings demonstrate that the application of automated lighting systems in the operating room presents a promising possibility for enhancing visibility during surgery and facilitating working conditions for medical personnel. In this paper, Virtual Reality assumes a central role, as a VR simulation was employed to assess the efficacy of an automated illumination system within the operating room. The VR simulation was executed on a computer, presenting an operating room-like scenario.

Reinschlüessel et al. delineate in their work the utilization of VR technology for the planning of liver tumor resections. Two instances of liver tumor resections are expounded upon, wherein VR was employed for surgical planning and preparation. The authors deliberate upon the advantages and challenges relating to the application of VR technology within the domain of surgery.

Staubli et al. outline a novel approach aimed at enhancing comprehension of biliary anatomy and facilitating surgical trainee education through the integration of Magnetic Resonance Cholangiopancreatography (MRCP) with VR. The authors have developed a VR application enabling users to visualize and manipulate biliary anatomy in a three-dimensional context. The application was subjected to case studies, demonstrating its capacity to augment the understanding of biliary anatomy and streamline the instructional process for surgical trainees.

Pu et al. discuss the use of endoscopic retrograde cholangiopancreatography (ERCP) combined with SpyGlass technology for the treatment of pancreatic duct stones (PDS). The authors present a case study of a 54-year-old male with severe abdominal pain and pancreatic duct stones. The study emphasizes the advantages of SpyGlass-assisted ERCP, including improved visualization, accurate biopsy, and successful stone removal, particularly for complex cases.

## Concluding remarks

The integration of immersive technologies such as AR, VR and MR into the field of medicine, specifically within the complex field of visceral surgery, unveils a vast array of potential opportunities that have the capacity to revolutionize patient care and everyday clinical practices across almost all domains. The studies of this Research Topic serve as the initial step towards a series of upcoming research projects vividly demonstrate the range of potential applications of extended reality techniques.

